# Neuroanatomical and Immunohistological Study of the Main and Accessory Olfactory Bulbs of the Meerkat (*Suricata suricatta*)

**DOI:** 10.3390/ani12010091

**Published:** 2021-12-31

**Authors:** Mateo V. Torres, Irene Ortiz-Leal, Andrea Ferreiro, José Luis Rois, Pablo Sanchez-Quinteiro

**Affiliations:** 1 Department of Anatomy, Animal Production and Clinical Veterinary Sciences, Faculty of Veterinary, University of Santiago de Compostela, Av. Carballo Calero s/n, 27002 Lugo, Spain; Mateovazquez.torres@usc.es (M.V.T.); irene.ortiz.leal@usc.es (I.O.-L.); 2 Marcelle Nature Park, Outeiro de Rei, 27154 Lugo, Spain; servet@marcellenatureza.com (A.F.); servet.marcellenatureza@gmail.com (J.L.R.)

**Keywords:** meerkat, *Suricata suricatta*, Herpestidae, Carnivora, olfactory system, vomeronasal system, olfactory bulb, immunohistochemistry, lectins, G proteins, atypical glomeruli

## Abstract

**Simple Summary:**

In wild mammals, chemical senses are crucial to survival, but sensory system information is lacking for many species, including the meerkat (*Suricata suricatta*), an iconic mammal with a marked social hierarchy that has been ambiguously classified in both canid and felid families. We studied the neuroanatomical basis of the meerkat olfactory and accessory olfactory bulbs, aiming to provide information on the relevance of both systems to the behaviors of this species and contributing to improving its taxonomic classification. The accessory olfactory bulb serves as the integration center of vomeronasal information. When examined microscopically, the accessory olfactory bulb of the meerkat presents a lamination pattern more defined than observed in dogs and approaching the pattern described in cats. The degree of lamination and development in the meerkat main olfactory bulb is comparable to the general pattern observed in mammals but with numerous specific features. Our study supports the functionality of the olfactory and vomeronasal integrative centers in meerkats and places this species within the suborder Feliformia. Our study also confirms the importance of chemical signals in mediating the social behaviors of this species and provides essential neuroanatomical information for understanding the functioning of their chemical senses.

**Abstract:**

We approached the study of the main (MOB) and accessory olfactory bulbs (AOB) of the meerkat (*Suricata suricatta*) aiming to fill important gaps in knowledge regarding the neuroanatomical basis of olfactory and pheromonal signal processing in this iconic species. Microdissection techniques were used to extract the olfactory bulbs. The samples were subjected to hematoxylin-eosin and Nissl stains, histochemical (*Ulex europaeus* agglutinin, *Lycopersicon esculentum* agglutinin) and immunohistochemical labelling (Gαo, Gαi2, calretinin, calbindin, olfactory marker protein, glial fibrillary acidic protein, microtubule-associated protein 2, SMI-32, growth-associated protein 43). Microscopically, the meerkat AOB lamination pattern is more defined than the dog’s, approaching that described in cats, with well-defined glomeruli and a wide mitral-plexiform layer, with scattered main cells and granular cells organized in clusters. The degree of lamination and development of the meerkat MOB suggests a macrosmatic mammalian species. Calcium-binding proteins allow for the discrimination of atypical glomerular subpopulations in the olfactory limbus between the MOB and AOB. Our observations support AOB functionality in the meerkat, indicating chemosensory specialization for the detection of pheromones, as identified by the characterization of the V1R vomeronasal receptor family and the apparent deterioration of the V2R receptor family.

## 1. Introduction

The dense network of chemosignals recognized by the olfactory system enables the animal to form a multidimensional rendering of its environment [1], information often used to modulate basic activities, such as reproduction, food-seeking, and the adoption of social, maternal, and sexual behaviors [2]. This environmental rendering is not static but dynamic in space and time. The physicochemical characteristics of detected signals, such as volatility and permanence in time and dispersion in space, can enhance the amount of information provided by each signal [3]. Identifying a diverse plethora of molecules associated with such a diverse range of solubilities and volatilities requires a great diversity of chemoreceptors, which have been identified phylogenetically in the olfactory rosette of fish [4] and are highly conserved and expanded in mammals [5].

The nasal cavity harbors several olfactory subsystems [6,7], including two sophisticated nasal chemosensory organs: the main olfactory epithelium (MOE) and the vomeronasal organ (VNO). Both organs are distinguished by the anatomical distributions of their sensory neurons, the types of receptors they express, the signaling mechanisms they employ to transduce chemosensory stimuli, the types of chemosensory stimuli they detect, and the axonal targets of their sensory neurons in the rhinencephalon [8].

The main olfactory system (MOS) is composed of ciliated olfactory sensory neurons (OSNs) located in the MOE lining the ethmoid turbinates and the main olfactory bulb (MOB), which represents the first integrative center for the information delivered by OSNs. The MOS is a broadly tuned odor sensor that responds to thousands of volatile chemicals carrying information on the state of food, detects pathogens, and identifies the presence of prey, predators or conspecifics [9]. Each OSN expresses a single G protein-coupled olfactory receptor (OR) from a pool of hundreds. All axons expressing the same OR, despite being randomly distributed in the MOE, project onto one or a few topographically invariant glomeruli on each side of the MOB [10].

The vomeronasal system (VNS) consists of microvillar vomeronasal sensory neurons in the neuroepithelium of the VNO [11] and the accessory olfactory bulb (AOB), where these neurons project [12]. The vomeronasal neurons are narrowly tuned sensory neurons that detect a range of small, natural ligands present in the exocrine secretions of conspecifics. The VNS is essential for the detection of both kairomones [13,14] and nonvolatile pheromones, which are involved in maternal recognition [15] and nonconscious sociosexual behaviors [16,17]. VNO neurons expressing the same vomeronasal receptor (VR) project axons to 10 to 30 distinct AOB glomeruli [18,19]. Located in the dorsocaudal region of the olfactory bulb (OB) and much smaller than the MOB, the AOB is usually well-differentiated and functionally independent from the MOB [20].

Although both the MOS and VNS present a degree of overlap in the stimuli they detect and the effects that they mediate [21,22], the rapid evolution of receptor genes is specific to the VNS [23]. The high degree of divergence among VR genes [24] differs from the general pattern of evolutionary conservation observed among olfactory receptors in the MOS [25]. The molecular diversity of the VNS is also reflected by a wide range of morphological heterogeneity, especially in terms of size, lamination, and the presence or absence of the AOB [26], which can make the interspecies extrapolation of information concerning the anatomical and histological features of the VNS very risky [27].

The meerkat (*Suricata suricatta*) is a gregarious carnivore of the suborder Feliformia, which includes species such as cats, hyenas, mongooses, and civets. In the past, the meerkat was classified within the Viverridae family, but in 1993, the meerkat was reclassified into the Herpestidae family due to the identification of new genetic similarities identified between meerkats and Herpestidae [28]. Meerkats live in semiarid environments in despotic groups of 2 to 50 individuals [29]. The dominant pair monopolizes reproduction, leading to a high level of intrasexual conflict, particularly among females, as the dominant female commonly evicts the eldest subordinate and other females during the late stages of pregnancy [30]. Males are the dispersing sex, leaving their natal groups at approximately 2 years of age [31]. Although most members of the suborder Feliformia are considered carnivores, some Herpestidae, such as the meerkat, are omnivores. The meerkat diet is primarily composed of arthropods, small-bodied mammals, and other vertebrates [32].

The complex social organization of meerkats suggests a high degree of morphological development for the MOS and VNS; however, to our knowledge, no information is available regarding the neuroanatomy of either the vomeronasal or olfactory systems of the meerkat. Behavioral studies indicate that meerkats are able to use olfactory signals when foraging [33,34], and their sense of smell appears to be specialized for the recognition of specific sociosexually relevant odors. Similarly, olfactory signals appear to play crucial roles in suppressing subordinate reproduction [35], establishing territorial areas, maintaining mate bonds [36], and perpetuating the social hierarchy [37]. Exocrine secretions released from the meerkat anal sac provide chemical information regarding sex, age, and the hierarchy within the social group [38,39], allowing for discrimination between relatives and nonrelatives [40]. In addition to a wide repertoire of scent-marking behaviors [41], meerkats display a flehmen response [33], which is indicative of a functional VNS. Similarly, both wild and captive meerkats can distinguish feces derived from carnivores and herbivores based on olfactory signals [31] and use predator cues, such as urine, to assess the level of danger associated with specific circumstances [42].

The relevance of chemical communication in meerkats indicates the importance of obtaining accurate neuroanatomical information regarding the structures of the meerkat MOS and VNS, which can be contrasted with available information for domestic carnivores, such as the cat [43] and dog [44,45]; wild carnivores, such as the African wild dog [46]; and with species belonging to the order Pholidota, a sister order to Carnivora, such as the pangolin [47]. We performed a comprehensive morphological, histological, and immunohistochemical study of meerkats with the aim of characterizing the neuroanatomical features of the first neural integrative centers of olfactory and vomeronasal information, the OB, and its main and accessory components.

## 2. Materials and Methods

Through a collaboration with Marcelle Nature Park (Outeiro de Rei, Spain), we were provided with three adult male meerkats (*Suricata suricatta*) for use in this study. The samples were collected after the animals’ death due to natural causes. No more than 8 h elapsed between the death of the animals and their processing.

### 2.1. Sample Extraction

To perform the complete extraction of the brain, we began by making a longitudinal incision in the skin of the skull to facilitate the removal of the skin and underlying muscle planes. The cranial bones were then removed with the help of a rotatory saw and a gouge forceps, leaving the cerebral hemispheres accessible. The next step was the careful removal of the olfactory bulbs from their hidden location in the ethmoidal fossa. These are particularly delicate, and additionally, the olfactory and vomeronasal nerves, which pass through the lamina cribrosa of the ethmoid, anchor them firmly to the dura mater. The bony walls of the fossa were removed while the olfactory nerves were severed. Then the optic nerves, the internal carotid arteries, the pituitary stalk and the cranial nerves that emerge on either side of the brainstem were cut leaving the brain free. The whole brain was fixed with freshly prepared Bouin’s liquid. Bouin’s fixative is especially recommended for the study of the nervous system due to its high tissue penetration and because it preserves the structure of soft and delicate tissues [48], which facilitates further processing. After 24 h, the samples were introduced in 70% alcohol. This procedure was followed in two of the animals. In the third one, the sample was directly fixed in formaldehyde, the nasal cavity was cut transversely to observe the topography of the vomeronasal organ, and the anterior part of the skull was removed so that, after removing the frontal lobes, the olfactory bulbs could be examined in situ.

### 2.2. Sample Processing for Histological Study

The olfactory bulbs were embedded in paraffin, cut in a rotary microtome at 8 μm in thickness and mounted onto gelatin-coated slides. The whole olfactory bulbs were serially sectioned in both sagittal and transversal planes. This allowed us to identify the much smaller accessory olfactory bulb. To visualize the different tissue components, we employed hematoxylin-eosin (HE) as a general staining and Nissl stain to visualize the soma and the beginning of the neuronal processes. In both specimens the results were comparable both structurally and in terms of immunohistochemical pattern.

### 2.3. Immunohistochemical Labelling (IHQ)

The immunohistochemical study covered a selected number of antibodies, which provide useful information on both MOS and VNS morphofunctional features. Antibodies against Gαi2 and Gαo allowed us to determine which pheromone receptor families—V1R [49] or V2R [50], respectively—are expressed in the VNS. The mitral cells, primary neural elements of the AOB, were labelled with antibodies against the microtubule-associated protein 2 (MAP-2) [51] and against SMI-32, marker of neurofilament proteins [52] expressed in the mitral cells of the OB of some therian mammals [53]. Neuronal growth was studied by employing antigrowth-associated protein 43 (GAP-43) [54]. The maturity of the system was determined using anti-olfactory marker protein (OMP) [55]. The calcium-binding proteins calbindin (CB) and calretinin (CR) were used to identify neuronal subpopulations [56]. Astrocytes and ensheathing cells were identified by an antibody against glial fibrillary acidic protein (GFAP) [57].

After deparaffining and rehydrating the samples, the procedure was as follows: (i) The slides were left for 10 min in a 3% hydrogen peroxide solution in distilled water. (ii) Nonspecific unions were blocked in the normal horse serum included in the ImmPRESS Anti-Rabbit and Anti-Mouse Kit from Vector Labs (Burlington, NJ, USA). Subsequently, (iii) the primary antibody was added at the corresponding dilution and the preparations were incubated overnight in a humid chamber at 4 °C. In any case antigen retrieval procedures were performed. The following morning, (iv) the corresponding secondary antibody (ImmPRESS Kit; view Table 1) was incubated for 30 min. Finally, (v) after rinsing in buffer (pH 7.6) for 10 min, (vi) the reaction was visualized by incubation in a 0.05% 3,3′-diaminobenzidine (DAB) solution and a 0.003% H_2_O_2_ solution, in 0.2 M Tris-HCl buffer. DAB chromogen developed into a brown precipitate. For several of the antibodies employed, counterstaining was performed with haematoxylin.

In all the immunohistochemical procedures, samples for which the primary antibody was omitted were used as negative controls, without obtaining labelling or unspecific background in any case. As positive controls, we replicated the immunohistochemical procedure with mouse tissues known to express the proteins of interest, obtaining the expected positive results.

### 2.4. Histochemical Labelling (HQ) with Lectins

Lectin-histochemical labelling is based on the use of the bond established between agglutinins, called lectins, with carbohydrates that form glycoconjugates in tissue components.

For this study we used three lectins: UEA (*Ulex europeaus* agglutinin), LEA (*Lycopersicon esculentum* agglutinin), and BSI-B_4_ (*Bandeiraea simplicifolia* isolectin B_4_). UEA comes from gorse and is able to bind the L-fucose presents in both glycolipids and glycoproteins [58]. This lectin specifically labels the canid vomeronasal system [59]. LEA from tomato has affinity towards N-acetylglucosamine [60] and is a specific marker for both olfactory systems [61]. BSI-B_4_ is a lectin with an affinity for terminal a-galactose extracted from the seeds of the legume shrub *Griffonia simplicifolia* [62] that is specific for the VNS in both rats [63] and opossums [64].

#### 2.4.1. LEA and BSI-B_4_ HQ Protocol

Biotinylated conjugates of LEA and BSI-B_4_ were used for this study (see details in Table 1). (i) We used 3% hydrogen peroxide (H_2_O_2_) solution to suppress the endogenous peroxidase activity. (ii) After two washes in 0.1 M phosphate-buffered (pH 7.2) solution (PB), (iii) the sections were incubated for 30 min in a 2% bovine serum albumin (BSA) in PB. (iv) Slides were then incubated overnight at 4 °C with LEA and BSI-B_4_ lectins, independently, in a 0.5% BSA solution. (v) After 2 washes in PB, (vi) they were incubated during 1.5 h at room temperature with avidin-biotin-peroxidase complex (ABC) reagent (Vectastain; Vector Laboratories, Burlingame, CA, USA). (vi) The slides were developed in the same way as the slides in the IHQ protocol.

#### 2.4.2. UEA-I HQ Protocol

As with the LEA and BSI-B_4_ lectins, after deparaffinizing and rehydrating the slides (i) they were incubated in 3% H_2_O_2_ solution. (ii) Then, they were incubated for 1 h at room temperature in a 0.5% BSA/UEA-I solution and (iii) then they were washed for 3 × 5 min in a PB solution. (iv) The slides were then incubated overnight at 4 °C with an anti-UEA-I peroxidase-conjugated antibody. (v) The next day, the samples were washed with a PB solution, and (vi) a DAB solution was added to visualize the reaction, as described for the LEA and BSI-B_4_ lectins.

Controls were performed for both protocols, both without the addition of lectins and with the preabsorption of lectins, by using an excess amount of the corresponding sugar.

### 2.5. Acquisition of Images and Digital Treatment

Pictures were taken with a Karl Zeiss Axiocam MRc5 digital camera coupled to a Zeiss Axiophot microscope. If necessary, Adobe Photoshop CS4 (Adobe Systems, San Jose, CA, USA) was used to adjust parameters such as brightness or contrast, balance light levels, and crop or resize images for presentation in this work. No specific characteristics within the images were altered, enhanced, moved or introduced. Some photomicrographs were formed as a mosaic of several pictures merged with two different image-stitching softwares (PTGui Pro and AutoStich).

## 3. Results

### 3.1. Macroscopic Study

**Figure 1:**
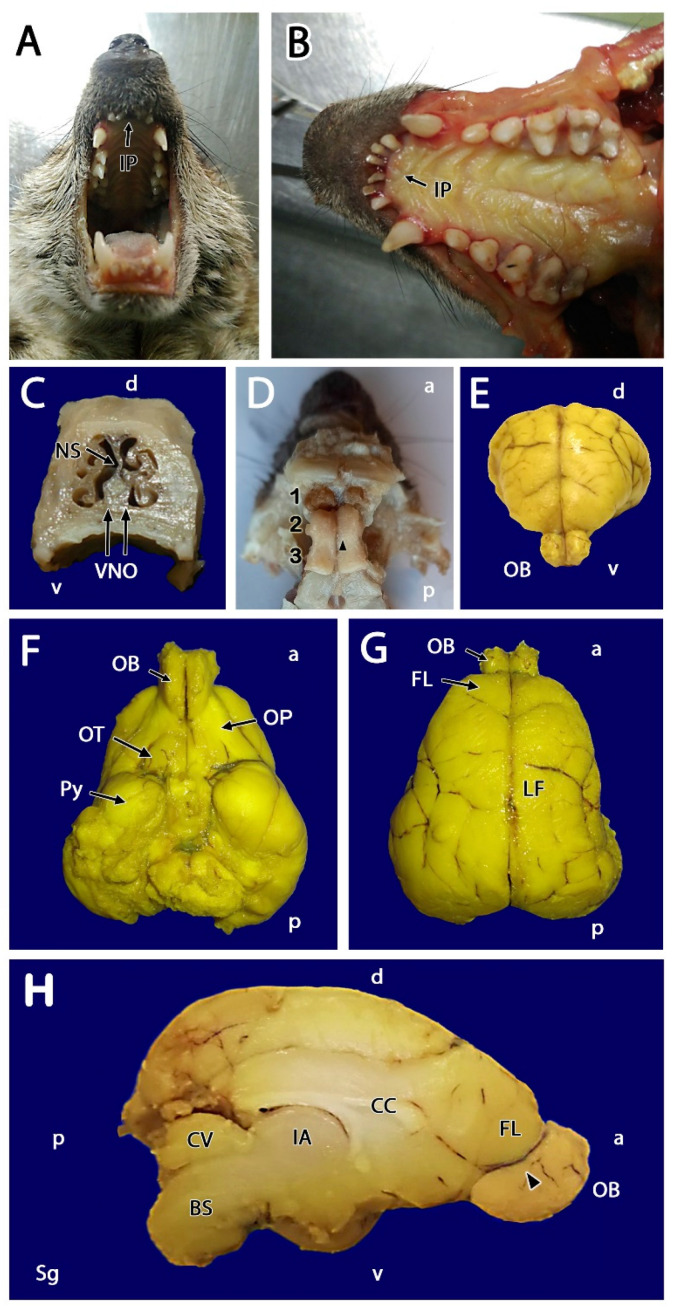
Main macroscopic features of the vomeronasal system of the meerkat. (**A**) Cranioventral view of the palate allows the identification of the incisive papilla (IP) area. (**B**) The IP is visible after the removal of the mandible. (**C**) Transverse section of the nasal cavity at the level of the first premolar, showing the vomeronasal organ (VNO) at the base of the nasal septum (NS), on both sides of the vomer bone (right). (**D**) Dorsal view of a dissection of the OB. The skull has been removed and the frontal lobe has been extracted. The presumptive area of localization for the right accessory olfactory bulb is indicated (arrowhead). 1, Ethmoidal conchae; 2, Olfactory bulb; 3, Olfactory peduncle. (**E**) In the rostral view of the brain is visible the development of the olfactory bulbs (OB) and their topographical relationship with the frontal lobe of the telencephalon. (**F**) Ventral view of the brain showing the olfactory system of the meerkat: olfactory bulb (OB), olfactory peduncle (OP), olfactory tubercle (OT), and pyriform lobe (Py). (**G**) Dorsal view of the brain showing the longitudinal fissure of the telencephalon (LF) as well as the arrangement and relative size of the olfactory bulbs. (**H**) Medial view of the left hemiencephalon showing the following anatomical structures: corpus callosum (CC), interthalamic adhesion (IA), brainstem (BS), cerebellar vermis (CV), frontal lobe (FL), and olfactory bulb (OB). The black arrowhead points to the presumptive area of localization of the accessory olfactory bulb. a, anterior; d, dorsal; p, posterior; v, ventral.

Prior to the dissection of the meerkat OB, both the oral and nasal cavities were examined to identify the main topographical landmarks of the VNS, as the VNS had not been previously described in this species. The incisive papilla (IP) represents an important access point through which pheromones reach the VNO, consisting of an elevation of the mucosa at the roof of the palate into which the nasopalatine duct opens. Also known as the incisive duct, the IP establishes direct communication with the vomeronasal duct. After separating the jaws (Figure 1A), the masticatory muscles were sectioned, and the mandible was dislocated at the level of the temporomandibular joint to facilitate access to the palatal mucosa (Figure 1B). The IP is located in the anterior region of the soft palate, caudal to the upper incisor teeth. The skin and muscle layers covering the skull and face were then removed. The cross-sectioning of the nasal cavity at the level of the first premolar allowed us to appreciate the development of the nasal septum, the dorsal and ventral nasal turbinates, and the presence of the VNO, which is located in the ventral region of the nasal septum on both sides of the vomer bone (Figure 1C).

After removing the anterior part of the skull in one animal and dissecting out the frontal lobe, the dorsal area of the OB could be observed in situ, with no evidence, beyond a minimal dorsomedial elevation, of the presumed AOB (Figure 1D). After extracting the entire intact encephalon from two other specimens, the OB was found to be well-developed and kidney-shaped (Figure 1E–H). The remaining olfactory structures that comprise the rhinencephalon were visible in a ventral view: the olfactory peduncle, olfactory tubercle, and piriform lobe (Figure 1F). After separating both hemiencephalons at the longitudinal fissure (Figure 1G), the medial aspect of the OB was visible in greater detail (Figure 1H); however, the AOB could not be identified. A very small protuberance on the posterior third of the medial edge of the MOB was noted, which was presumed to be the location of the AOB (arrowhead in Figure 1H), an assumption that was later confirmed by histological serial sectioning of the whole OB.

### 3.2. Microscopical Study

#### 3.2.1. Histological Study

**Figure 2:**
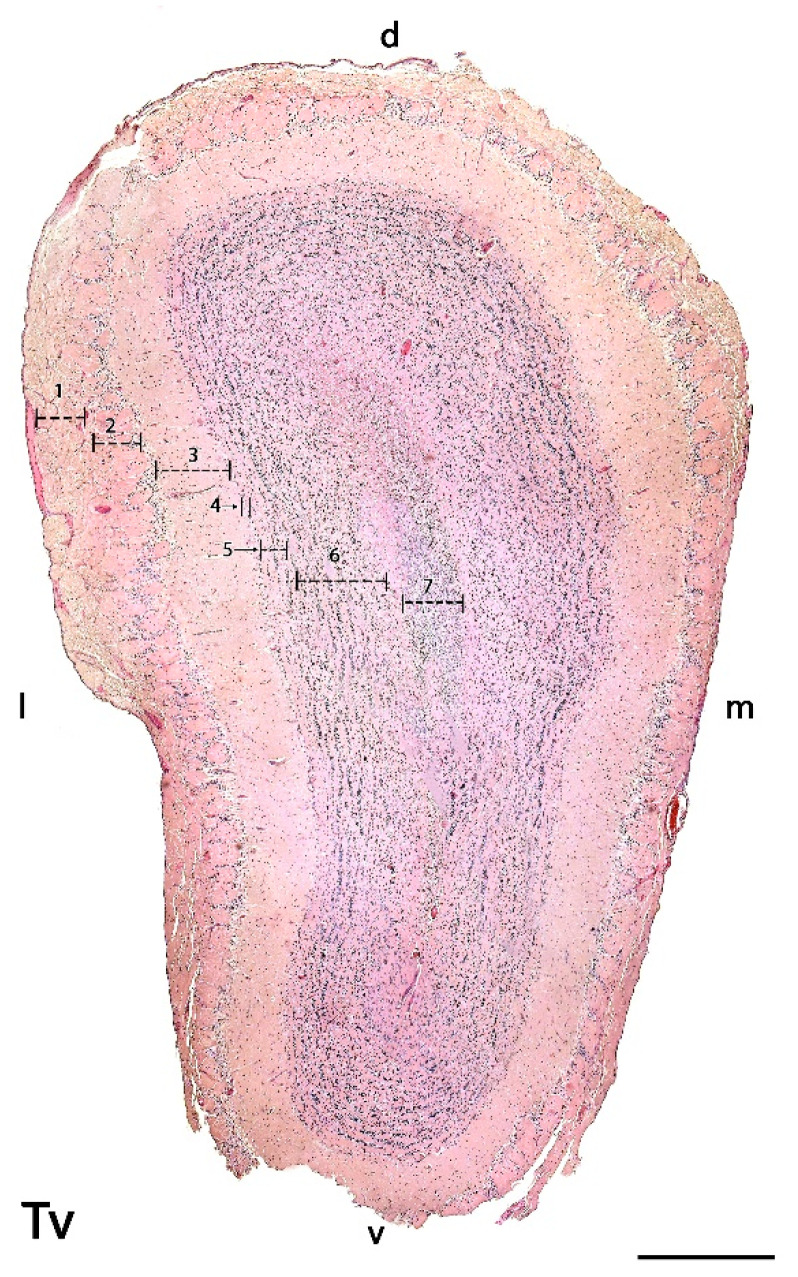
Transverse section of the main olfactory bulb of the meerkat stained by hematoxylin-eosin. From superficial to deep the following layers are identified: 1. Olfactory nerve layer (ONL), 2. Glomerular layer (GlL), 3. External plexiform layer (EPL), 4. Mitral layer (ML), 5. Internal plexiform layer (IPL), 6. Granular layer (GrL), 7. Subventricular zone (SVZ). d, dorsal; l, lateral; m, medial; v, ventral. Scale bars: 600 µm. Tv, Transverse.

**Figure 3:**
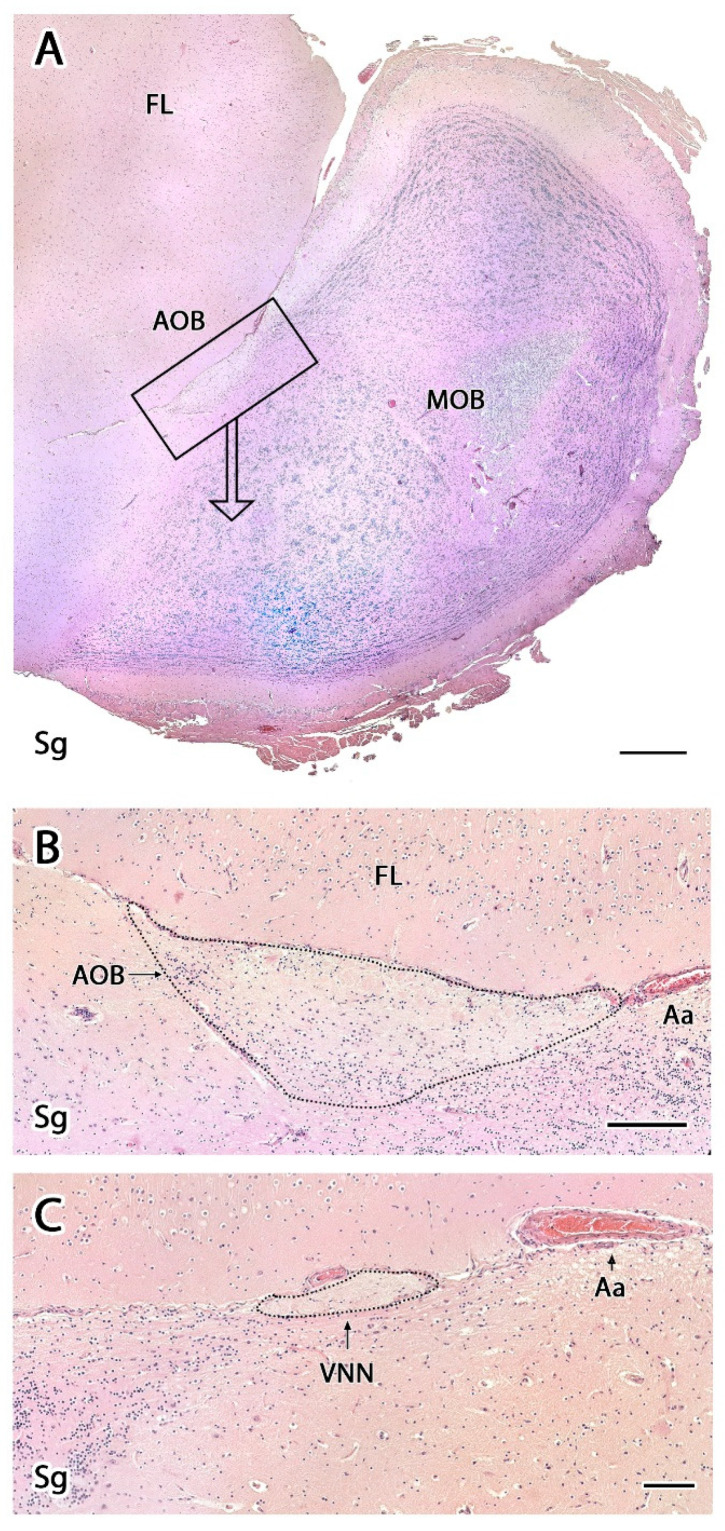
Sagittal sections of the olfactory bulbs of the meerkat stained with Hematoxylin-eosin. (**A**) Sagittal section of the rostral portion of the brain of the meerkat. The frontal lobe (FL), the main olfactory bulb (MOB), and the location, in the caudal part of the MOB, of the accessory olfactory bulb (AOB) are shown. (**B**) Higher magnification of the box in (**A**) showing the AOB. Following the course of the vomeronasal nerve throughout the sagittal histological series, we arrived at the area bordered by the dotted line that corresponds to the AOB. Its size is relatively small and its lamination, diffuse. It is better discriminated with the Nissl stain (Figure 4 and Figure 5). (**C**) A more medial sagittal section than B shows the arrival of the vomeronasal nerve through the medial surface of the main olfactory bulb in direct topographical relationship to a large-caliber artery (Aa). Scale bars: (**A**): 600 µm. (**B**): 200 µm. (**C**): 100 µm. VNN, vomeronasal nerve, Sg, Sagittal.

The serial microscopic study of the OB with hematoxylin-eosin (HE) and Nissl stains confirmed that the majority of the OB located at the rostral aspect of the brain is occupied by the MOB (Figure 2 and Figure 3A). The existence of an AOB in the meerkat was also indicated by the microscopic study, although the small size of the AOB prevented its localization by macroscopic dissection. The microscopic study also allowed for the determination of the degree of lamination compared with the MOB, which is structurally organized following a pattern that is common among mammalian species.

We were able to easily identify the typical seven-layer structure of the MOB in both transverse (Figure 2) and sagittal (Figure 3A) sections. Moving from the superficial to deep planes, these layers include:

1. Olfactory nerve layer (ONL): Formed by the axons of the olfactory nerves that reach the MOB.
2. Glomerular layer (GlL): Composed of the glomeruli, which are spherical structures delimited by periglomerular (PG) cells that originate from the confluence of olfactory nerve axons synapsing with the dendrites of mitral cells, which represent the second neurons in olfactory pathways.
3. External plexiform layer (EPL): A nerve plexus with a low cell density, primarily occupied by the dendrites of mitral cells.
4. Mitral cell layer (MCL): Formed by the somas of mitral cells.
5. Internal plexiform layer (IPL): A thin band of white matter interposed between the mitral and granular layers that is very reduced in the meerkat.
6. Granular layer (GrL): Contains large clusters of granule cells, which serve as inhibitory neurons in the neural circuit of the MOB.
7. Subventricular zone (SVZ): Nervous tissue arranged around the rostral horn of the encephalic lateral ventricles, primarily composed of neuronal precursor cells involved in adult neurogenesis. The SVZ in meerkats is highly and strikingly developed, presenting a large cellular infiltrate.

**Figure 4:**
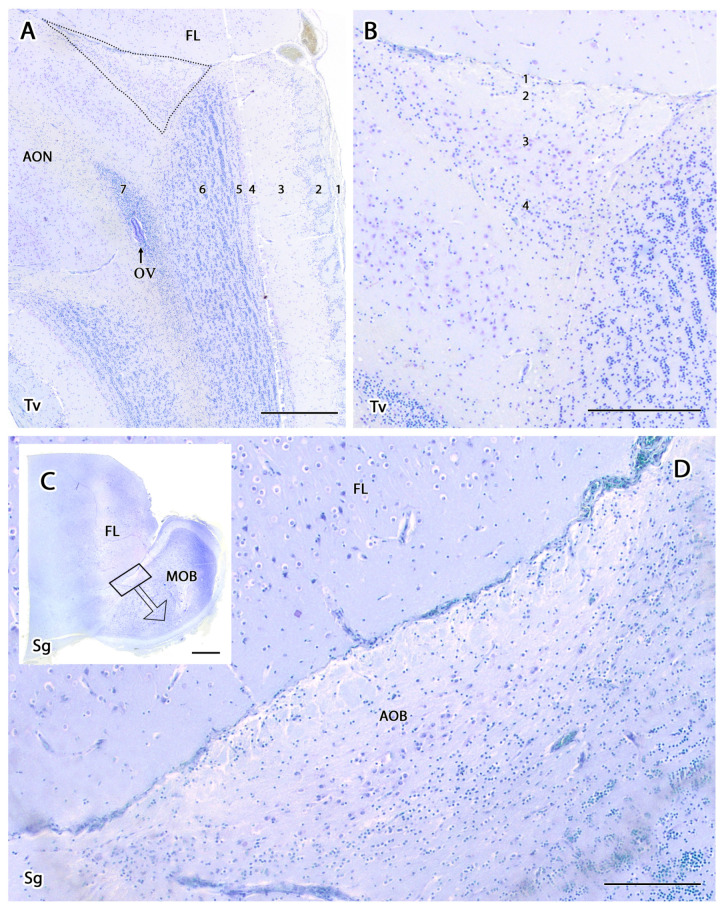
Histological study of the accessory olfactory bulb of the meerkat with Nissl staining. (**A**) Transverse section of the olfactory bulb showing the projection area of the accessory olfactory bulb (AOB) (delimited by dots), the lamination of the main olfactory bulb and the anterior olfactory nucleus (AON). The numbering of the layers is identical to that employed in Figure 2. (**B**) Enlargement of the AOB shown in A with its different layers numbered. 1, Vomeronasal nerve layer; 2, Glomerular layer; 3, Mitral-plexiform layer; 4, Granular layer. (**C**) Sagittal section of the olfactory bulb and the frontal lobe (FL) of the telencephalon showing the lamination of the main olfactory bulb (MOB) and the presence of the AOB in its caudomedial part. (**D**) Enlargement of the box in (**C**) showing the histology of the AOB. OV, olfactory ventricle. Scale bars: (**A**) 400 µm; (**B**) 200 µm; (**C**) 1200 µm; (**D**) 150 µm.

**Figure 5:**
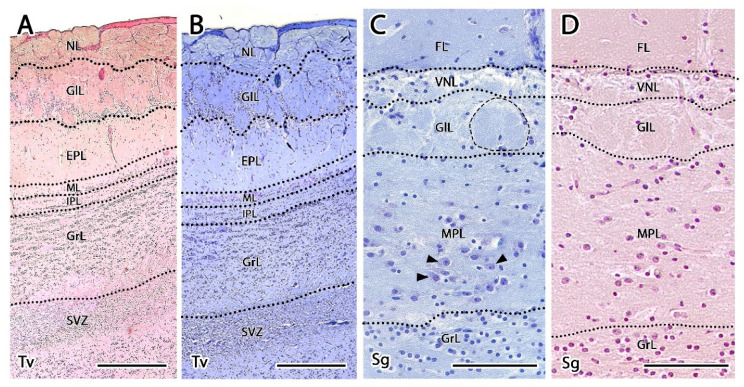
Histological comparative study of the lamination of both the main and accessory olfactory bulbs. Lamination of the main olfactory bulb showed with HE (**A**) and Nissl staining (**B**): olfactory nerve layer (NL), glomerular layer (GlL), external plexiform layer (EPL), mitral layer (ML), internal plexiform layer (IPL), granular layer (GrL), subventricular zone (SVZ). The lamination of the accessory olfactory bulb is shown with HE (**C**) and Nissl staining (**D**): vomeronasal nerve layer (VNL), glomerular layer (GlL), mitral-plexiform layer (MPL) and granular layer (GrL). The small degree of differentiation of the accessory olfactory bulb glomeruli is mainly due to the scarcity of periglomerular cells. Only in a few cases (dotted line) is the usual spherical shape visible. The main cells of the AOB are diffusely distributed along the mitral-plexiform layer, especially in its deepest zone. They are oval or polyhedral in shape (black arrowhead). Granular cells are smaller and distributed in irregular clusters. Front lobe of the telencephalon (FL). Tv: transversal plane, Sg: sagittal plane. Scale bars: (**A**–**D**): 100 µm.

The sagittal microscopic series (Figure 3A) allowed us to confirm the lamination of the MOB observed in the transverse section and facilitated the location at which the vomeronasal nerves (VNNs) enter the brain (Figure 3C), together with an artery of remarkable size. This region corresponds to the AOB, which is located in the caudomedial zone of the MOB, in a direct topographic relationship with the frontal lobe of the telencephalon. The meerkat AOB is characterized by small size and a low degree of differentiation among its layers (Figure 3B). Nissl staining (Figure 4 and Figure 5) is particularly useful for characterizing the cellular elements and layers that comprise the AOB. In both transverse (Figure 4A,B) and sagittal sections (Figure 4C,D), the low degree of cellularity in the AOB compared with the MOB is striking, making the differentiation of the AOB from the MOB challenging, particularly in the sagittal plane. To understand the peculiar organization of the AOB in the meerkat, a comparative study of the lamination was performed between the MOB and AOB, as shown in Figure 5. The complex histological structure of the MOB is reduced to the following four layers in the AOB:

1. Vomeronasal nerve layer (VNL): formed by the arrival of VNN axons.
2. Glomerular layer (GlL): glomeruli in the AOB show poorer definition and fewer PG cells than their MOB equivalents (Figure 5C,D).
3. Mitral-plexiform layer (MPL): the cells of the mitral layer are not organized in a monolayer, as observed in the MOB (Figure 4A,B), but are diffusely distributed, occupying a large neuropil due to the fusion of the external and internal plexiform layers with the mitral layer, forming what we term the MPL. The main cells found in this layer present an ovoidal morphology, reminiscent of the morphology of mitral cells in the MOB (Figure 5C,D).
4. Granular layer (GrL): presenting with a higher cell density, the neurons in this layer have small nuclei and compact cytoplasms (Figure 5C,D).

**Figure 6:**
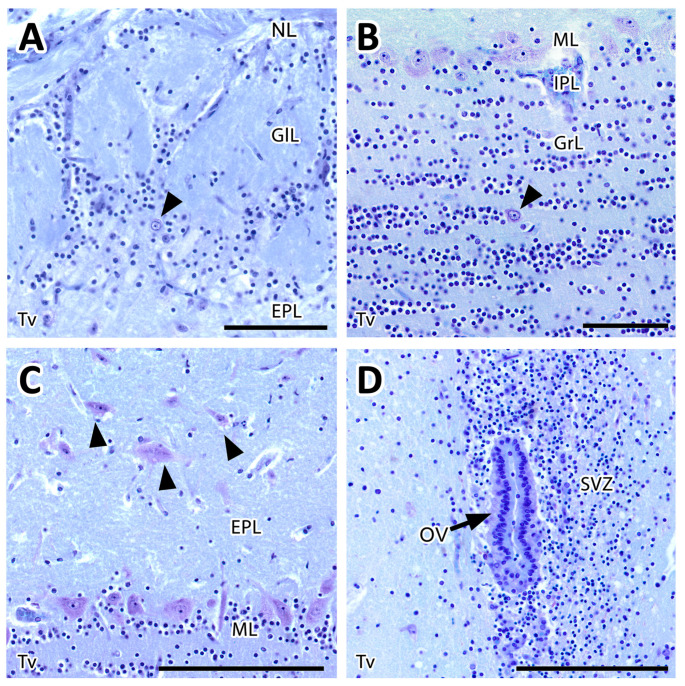
Histological study with Nissl stain of the structure of the main olfactory bulb. (**A**) The glomerular layer (GlL) comprises the typical spherical glomeruli surrounded by numerous periglomerular cells. Scattered neurons with large somas are also found (arrowhead). (**B**) The granular layer (GrL) is formed by neat clusters of granule cells interspersed with large neuronal somas (arrowhead). (**C**) Mitral cells somas are aligned along the mitral layer (ML), whereas the external plexiform layer (EPL) contains tufted cells (arrowheads). (**D**) The deep area of the MOB contains the rostral horn of the lateral ventricle, the olfactory ventricle (OV), with its ependyma (arrow) and a dense proliferative zone characteristic of the subventricular zone (SVZ) neuroprogenitor cells. NL, nerve layer. Scale bars: (**A**,**B**) 50 µm; (**C**,**D**) 100 µm.

Compared with the AOB, the cytological study of the MOB shows more diverse cellularity. The GlL is formed by typical, large, spherical glomeruli surrounded by numerous PG cells (Figure 6A), forming a broad band in the boundaries with the deeper layer. This band contains scattered neurons with large somas. The GrL is organized into large clusters of granule cells, interspersed with large neuronal somas (Figure 6B). Mitral cell somas are ordered along the MCL, forming a monolayer, whereas the EPL contains tufted cells (Figure 6C). The deeper area of the MOB contains the rostral horn of the lateral ventricle, known as the olfactory ventricle, with associated ependyma and the dense proliferative zone characteristic of the SVZ (Figure 6D).

#### 3.2.2. Immunohistochemical Study

**Figure 7:**
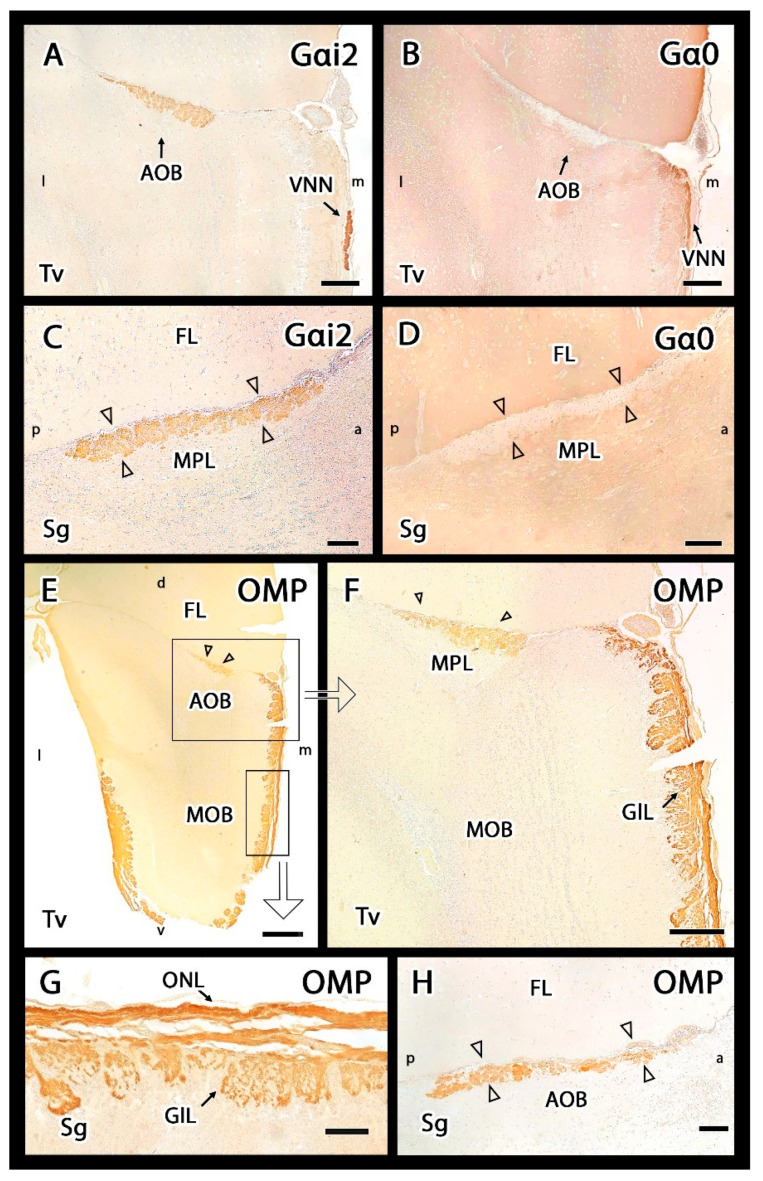
Immunohistochemical study of Gαo, Gαi2 and OMP proteins in the main and accessory olfactory bulbs of the meerkat. (**A**,**C**) Anti-Gαi2 immunolabels specifically the accessory olfactory bulb (AOB). Transverse section (**A**) and hematoxylin counterstained sagittal section (**C**) show strong immunopositivity confined to the vomeronasal nerve and glomerular layers of the AOB (arrowheads in (**C**)) as well as in the vomeronasal nerve (VNN in (**A**)). (**B**,**D**) Anti-Gαo immunolabelling in transverse and sagittal sections of the OB consecutive to the previous one showing a positive response complementary to that described for anti-Gαi2. That is, labelling in the whole main olfactory bulb (MOB) and both the mitral-plexiform (MPL) and granular layers of the AOB and absence of labelling in the superficial layers of the AOB (arrowheads in (**D**)). The VNN is not immunolabelled. (**E**–**H**) Anti-OMP immunolabelling. (**E**) Transverse section of the whole OB shows immunoreactivity in the superficial layers of both the MOB and AOB. (**F**) Enlargement of the upper box in (**E**). The immunostaining is confined to the nerve and glomerular layers. (**G**) Enlargement of the lower box in (**E**). Within the glomeruli, only the nervous component is labelled. (**H**) Sagittal section of the AOB showing at more magnification how the immunolabelling comprises the nerve and glomerular layers (arrowheads). a, anterior; d, dorsal; l, lateral; v, ventral; m, medial; p, posterior; Sg, sagittal; Tv, transverse. Scale bars: (**A**,**C**,**F**,**H**) 200 µm; (**B**,**D**,**G**) 100 µm; (**E**) 400 µm.

The Gαo and Gαi2 subunits of G proteins are useful markers for characterizing the expression of the two main VR families, V1R and V2R, in the AOB. Anti-Gαi2 antibodies revealed strong positive labelling in the AOB and the VNNs (Figure 7A). The VNL and GlL showed immunopositivity within the AOB, whereas the MPL and GrL were immunonegative (Figure 7C). The immunopositivity of the two superficial layers is due to the expression of VR proteins in VNN axons, which are distributed along the AOB surface and form the VNL, and at their axon terminals, which are found in the GlL, where they synapse with the dendrites of the second neurons of the vomeronasal pathway. This labelling allowed us to immunohistochemically confirm the presence of the AOB in the meerkat in both sagittal and transverse sections, which corresponded to the structures identified by HE and Nissl staining (Figure 3 and Figure 4). The anti-Gαi2 antibody specifically labelled the AOB, without labelling any features of the MOB.

Immunolabelling with the anti-Gαo antibody showed a complementary pattern to anti-Gαi2 labelling. In the AOB, anti-Gαo was immunonegative in the VNL and GlL. However, the deeper layers, including the MPL and GrL were immunopositive (Figure 7B,D). This immunopositivity extended to the remaining nerve structures: the entire MOB and the frontal lobe of the telencephalon.

The anti-OMP antibody selectively labels the OMP protein, which is specifically expressed by mature olfactory and vomeronasal sensory neurons. OMP staining produced a generalized reaction in the superficial nerve layer and GlL of both the MOB and AOB in both VNNs and olfactory nerves (Figure 7E–H). The intensity of OMP immunostaining was stronger in the MOB than in the AOB.

**Figure 8:**
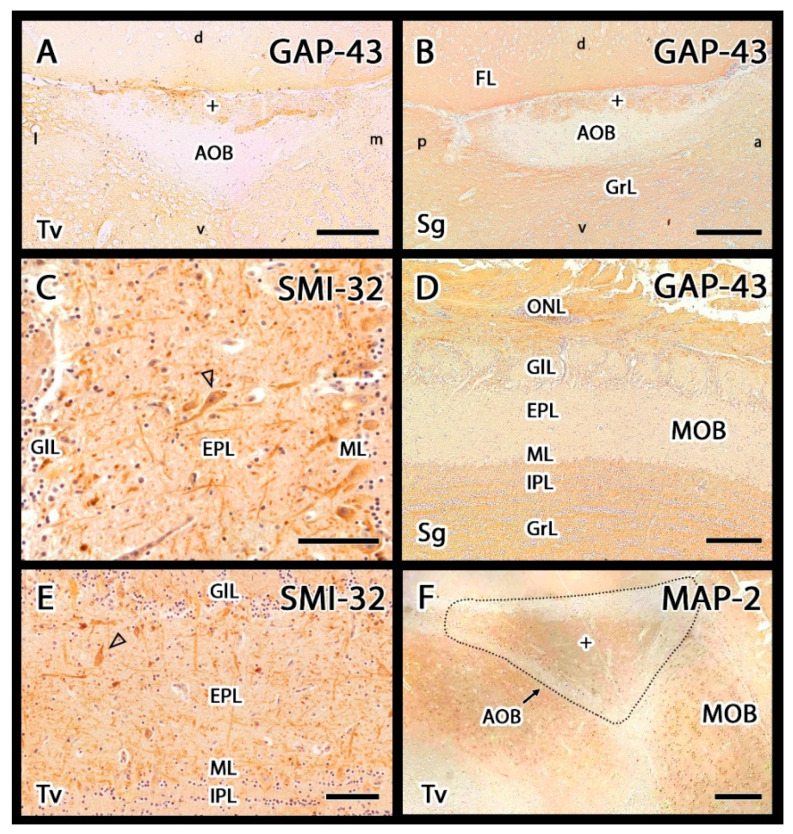
Immunohistochemical study of the olfactory bulb in meerkat (GAP-43, SMI-32 and MAP-2 markers). (**A**,**B**) Anti-GAP-43 produces in both transverse (**A**) and sagittal (**B**) sections a strong immunopositive labelling in the nerve, glomerular (+) and granular layers (Grl) of the AOB. (**D**) In the MOB Anti-GAP-43 marker showed immunopositivity in the ONL, IPL and GrL. (**C**,**E**) Anti-SMI-32 immunolabelling; hematoxylin counterstaining. This marker is immunonegative in the AOB, but it produces strong positivity in the principal cells of the MOB (arrowheads). (**F**) Anti-MAP-2 labels the mitral-plexiform and granular layers of the AOB (+) and the plexiform and mitral layers of the MOB. Sg: Sagittal; Tv, Transverse. Scale bars: (**A**,**C**,**E**,**F**) 100 µm; (**B**,**D**) 200 µm.

The GAP-43 antibody is typically used as a marker for axonal growth cones. In this study, GAP-43 positivity could be observed in the VNL, GlL, and GrL of the AOB, whereas the MPL was immunonegative (Figure 8A,B). In the MOB, GAP-43 immunopositivity was observed in the ONL, IPL, and GrL (Figure 8D). SMI-32 was present in the soma and dendrites of the principal MOB neurons, mitral, and tufted cells (Figure 8C,E). In the AOB, this antibody produced negative labelling. Anti-MAP-2 is an additional marker for the neural somas and dendrites. In both the MOB and AOB, MAP-2 labelling could be observed in the dendritic tree of principal cells, resulting in diffuse labelling of the mitral, plexiform, and granular layers (Figure 8F).

**Figure 9:**
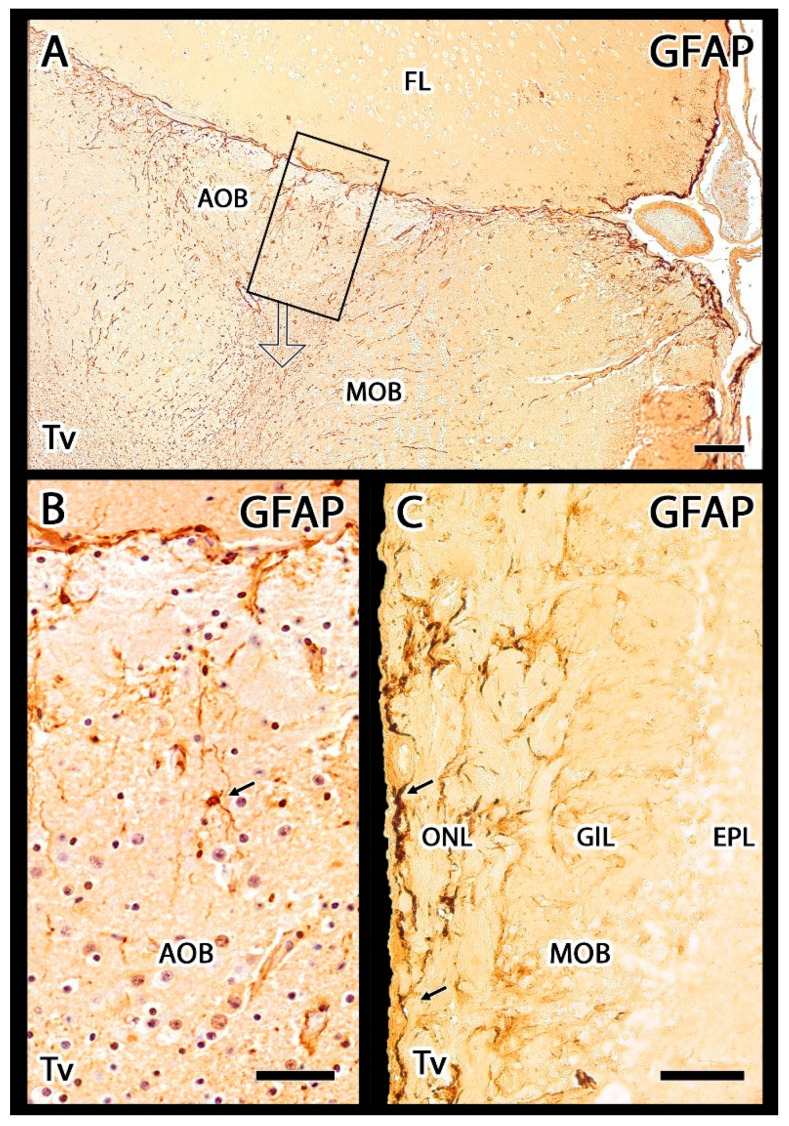
Immunohistochemical labelling of the meerkat OB with anti-GFAP. (**A**) Anti-GFAP produces a strong labelling in the nerve layer of both the accessory and (**C**) main olfactory bulbs. The labelling in these layers corresponds to glial cell fibers, mainly ensheathing cells. (**B**) Enlargement of the box in (**A**) after hematoxylin counterstaining, showing fibers and astrocytes in the mitral-plexiform layer of the AOB (arrow). (**C**) In the MOB there is a remarkable presence of astrocytes in the superficial layers (arrows). Scale bars: (**A**,**C**) 100 µm; (**B**) 50 µm.

Anti-GFAP produced immunolabelling in both the AOB and MOB. In the AOB, GFAP was primarily identified in the VNL (Figure 9A). The glial elements recognized by this marker primarily corresponded to the fibers of the ensheathing glia, although in the deeper layers of the AOB, some astrocytes could also be observed (Figure 9B). In the MOB, GFAP immunolabelling was concentrated in the astrocytes located in the superficial layers, and the deeper neuropil of the MOB and the frontal lobe were also immunopositive.

**Figure 10:**
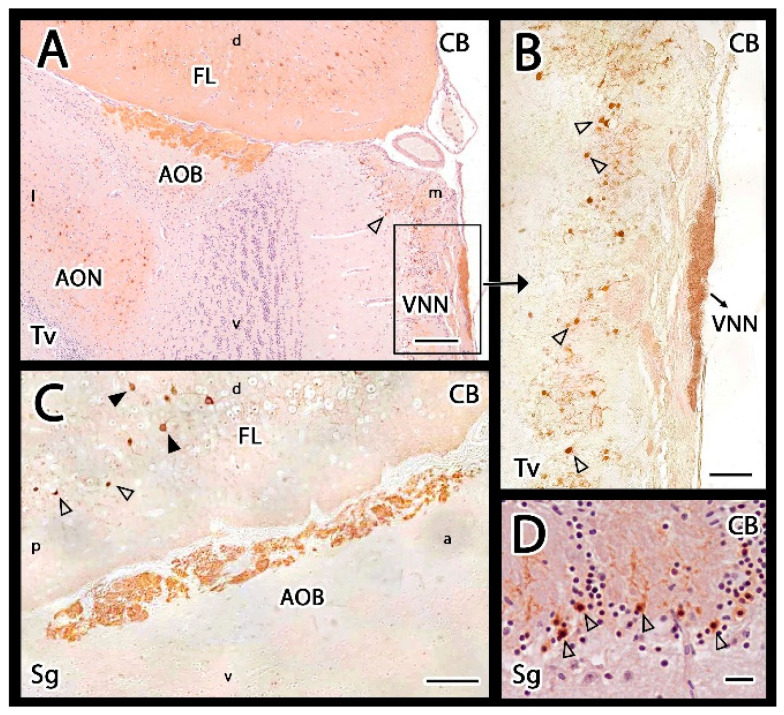
Immunolabelling with anti-calbindin (CB) in the olfactory bulb of the meerkat. (**A**) Transverse section of the olfactory bulb counterstained with hematoxylin showing strong immunopositivity to CB in the vomeronasal nerve (VNN), and in the neuropil of both the nerve and glomerular layers of the accessory olfactory bulb (AOB). Additionally, immunopositive neurons are observed in the cerebral cortex of the frontal lobe (FL), and the anterior olfactory nucleus (AON). (**B**) Enlargement of the inset in (**A**), not counterstained, showing the strong immunopositivity of the VNN (arrow) and the immunolabelling pattern of the main olfactory bulb (MOB). The arrowheads point to positive periglomerular cells belonging to the glomeruli of the MOB. (**C**) Sagittal section confirming the CB-immunopositivity of the AOB in its glomerular and nerve layers. Immunopositive cells with multipolar (black arrowheads) and granular (open arrowheads) morphology are observed in the FL. (**D**) Hematoxylin counterstaining in the glomerular layer of the MOB shows that only a subpopulation of periglomerular cells is CB-immunopositive (open arrowheads). Scale bars: (**A**,**C**) 100 µm; (**B**,**D**) 50 µm.

In both the transverse (Figure 10A,B) and sagittal (Figure 10C) sections of the OB, anti-CB antibody produced a very characteristic pattern, in which the VNNs of the VNL and GlL of the AOB were strongly stained, whereas the remaining AOB layers showed very weak staining. Although the glomeruli of the AOB were strongly stained, no positive staining was observed in the PG cells. By contrast, the study of anti-CB immunostaining in the MOB (Figure 10B) produced intense staining of the PG cells, whereas the glomerular neuropil showed very faint staining. Counterstained sections allowed for the determination that only a subpopulation of PG cells was CB-immunopositive (Figure 10D).

**Figure 11:**
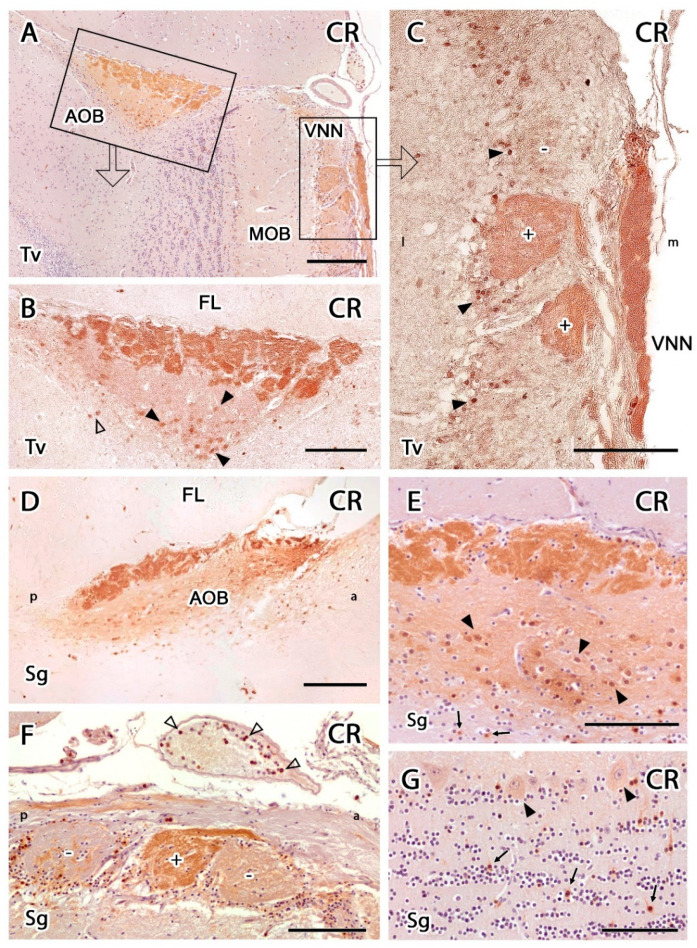
Anti-calretinin (CR) immunohistochemical study of the olfactory bulb in the meerkat. (**A**) Transverse section of the dorsomedial part of the olfactory bulb (OB) counterstained with hematoxylin. Strong immunopositivity can be seen in the accessory olfactory bulb (AOB), the vomeronasal nerve (VNN), and the glomeruli of the main olfactory bulb (MOB). (**B**) Enlargement of the left box in A, not counterstained. The immunolabelling of the AOB is concentrated in the nerve and glomerular layers and with less intensity in the mitral-plexiform and granular layers. Immunopositive mitral cells are identified in both the mitral-plexiform (black arrowhead) and granular layers (open arrowhead). (**C**) Enlargement of the area corresponding to the right box in A, but without counterstaining, showing the strong immunolabelling of the VNN and the presence in the dorsomedial part of the MOB of atypical CR-immunopositive glomeruli (+), in contrast to the typical CR-immunonegative glomeruli (−). A subpopulation of periglomerular cells were also immunopositive (arrowheads). (**D**) Sagittal section of the AOB confirming the positive immunolabelling pattern found in the transverse section. (**E**) Hematoxylin counterstained sagittal section of the AOB showing the density of immunopositive mitral cells in the mitral-plexiform layer (arrowheads) and isolated CR-positive granule cells in the granular layer (arrow). (**F**) Hematoxylin counterstained sagittal section of the MOB showing an atypical immunopositive glomerulus (+), surrounded by two immunonegative glomeruli (−). Immunopositive cells also appear in the artery accompanying the vomeronasal nerve (arrowheads). (**G**) Hematoxylin counterstained sagittal section of the MOB showing immunopositive mitral cells in the MOB (arrowhead). Scattered CR-immunopositive granule cells are interspersed among immunonegative granular clusters (arrows). FL, frontal lobe; Sg, sagittal; Tv, transversal. Scale bars: (**A**) 200 µm; (**B**–**F**) 100 µm; (**G**) 50 µm.

Anti-CR produced strong immunopositivity in the AOB, the VNNs, and the glomeruli of the MOB (Figure 11). Immunolabelling in the AOB was concentrated in the VNL and GlL, with some staining also observed in the mitral-plexiform and granular layers but to a lesser extent (Figure 11A,B). Whereas no immunolabelled cellular elements were observed in the superficial layers of the AOB, stained neurons could be observed in the MPL and GrL (Figure 11B,D,E), mainly immunopositive mitral cells. However, positive granule cells were scarce, and they tended to be isolated between immunonegative clusters (Figure 11E). Anti-CR immunolabelling of the MOB (Figure 11C,F,G) revealed a subpopulation of immunopositive PG cells (Figure 11C,F). Strikingly, the neuropil of most glomeruli in the MOB was negative, except for a subpopulation of atypical glomeruli in the vicinity of the AOB with strongly CR-positive neuropil (Figure 11C,F). In the deeper layers of the MOB, most of the mitral cell somas were immunopositive, as were isolated granule cells (Figure 11G). Additionally, CR-immunopositive cells were immunolabelled inside the vessels surrounding the VNN (Figure 11F).

#### 3.2.3. Lectin Histochemical Study

**Figure 12:**
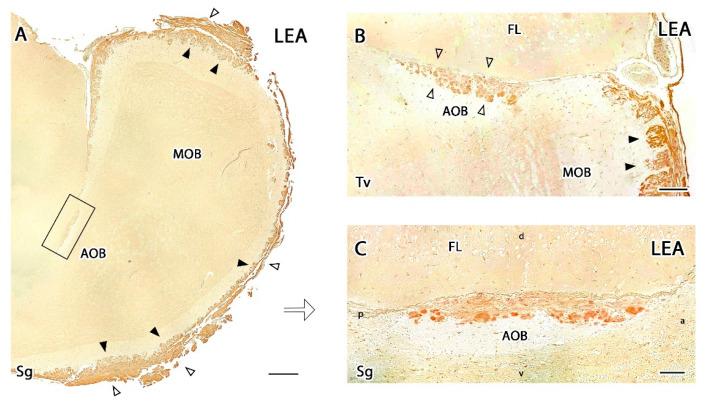
Histochemical study of the meerkat olfactory bulb with the lectin *Lycopersicum esculentum* (LEA). (**A**) Staining in the nerve (open arrowhead) and glomerular (black arrowhead) layers of the main olfactory bulb (MOB). Analogous layers of the accessory olfactory bulb (AOB) (box) show a less intense staining. (**B**) Transverse section of the olfactory bulb confirming the positive staining in the nerve and glomerular layers of both the AOB (open arrowheads), and MOB (black arrowheads). (**C**) Enlargement of the box in A showing LEA staining in the superficial layers of the AOB. Sg, sagittal plane; Tv, transverse plane. Scale bars: (**A**) 600 µm; (**B**) 100 µm; (**C**) 50 µm.

The histochemical study with *Lycopersicum esculentum* lectin (LEA) produced very intense staining in the glomerular and nervous layers of the MOB (Figure 12A), whereas the deeper layers did not show any positive labelling (Figure 12B,C). The AOB showed a similar pattern but was less intense, with LEA labelling concentrated in the VNN and GlL (Figure 12C). UEA-I and BSI-B_4_ staining did not result in any positive labelling in either the MOB or the AOB.

## 4. Discussion

The present study describes a comprehensive neuroanatomical and immunohistochemical characterization of the first integration center in the brain for information processed by the main and accessory olfactory systems of the meerkat. Despite the wide range of behaviors and physiological responses that have been described as mediated by chemical signals in this species, no neuroanatomical or immunohistochemical information was available for the meerkat OB. To our knowledge, no information for the OB is available for the entire Herpestidae family, except for a brief mention of dopaminergic PG neurons in the mongoose MOB within the context of a comprehensive study of the neurotransmitter systems found in the mongoose brain [65]. The adaptation of species to a very wide range of environments and the development of specific behavioral patterns has been associated with the distinct evolution of chemical communication systems and the diversification of the morphofunctional features of sensory organs and integrative nerve centers. These diverse adaptations can make extrapolations of neuroanatomical features between species challenging and inadvisable, even among species belonging to the same family, necessitating the performance of species-specific studies.

### 4.1. Neuroanatomical Study

In general terms, our observations indicate that the meerkat MOB displays the general cytoarchitecture pattern observed in all carnivores. However, the AOB presents important differences, which is not surprising considering the great morphological diversity that has been described for this structure in other mammals [25,66]. These differences likely represent adaptations to the numerous social and reproductive behavioral peculiarities associated with this species, many of which are mediated by chemical signals [67].

The lamination and cytoarchitectural patterns observed in the meerkat MOB do not differ significantly from those found in other Carnivora [43,44], nor do they differ from the patterns described in species belonging to other mammalian orders, including Primates, such as the macaque [68]; Artiodactyla, such as the hippopotamus [69] or whale [70]; Proboscidea, such as the elephant [71]; or Rodentia, such as mice [72]. The MOB of the meerkat shows remarkable development of the GlL, containing an abundant number of PG cells, particularly in the deep zone; a broad MPL rich in abundant, and the neat organization of perfectly aligned mitral cells. The granular cells of the meerkat MOB are organized into clusters, and the development of the SVZ is particularly striking, characterized by a very dense accumulation of cells. The SVZ of the meerkat is superior to the SVZ of the dog fetus OB [73], which is thought to serve as a more representative model than rodents for the types of neurogenic events that occur in humans [74]. The SVZ of the adult brain has the largest capacity for the constitutive regeneration of new neural cells [75,76]. The MOB serves as the final destination of new neural cells generated in the central neurogenic niches of the SVZ, which migrate through the rostral migratory stream to the OB [77,78].

Although the internal organization of the mammalian AOB is predominantly laminar, important structural and phylogenetic variations have also been described [26]. Whereas the AOB is fully developed in some groups, such as Rodentia [79], Marsupialia [80,81,82], Primates [83], and Artiodactyla [84], in other species, such as the African elephant [71], the West Indian manatee [85], and humans [86], the AOB is absent or has not yet been identified. In species such as the mink [87], ferret [88], and some bats [89], the AOB is present but shows little development, reflected by small size and a general lack of any typical cytoarchitecture without neatly differentiated layers [26].

Meerkats belong to the order Carnivora, and the AOB of the meerkat can be compared with other Carnivora species, such as canids and felids, whose VNS have been studied in-depth. The cat (*Felis silvestris catus*) and the dog (*Canis lupus familiaris*) provide striking examples of the great disparity in the degree of differentiation that can exist in the AOB. The cat AOB is well-developed, macroscopically appreciable and shows a clear definition of layers [90]. However, the low degree of differentiation observed in the dog AOB is surprising [59,91,92] and has long been considered a controversial issue because it contrasts with the important roles played by pheromone-based chemosensory communications [93] and the highly social behaviors attributed to this species [94,95]. In 1902, Ramón y Cajal [96] ruled out the existence of an AOB in the dog. However, Jawlowski (1956) [97] and Miodonski (1968) [98] subsequently identified a putative AOB in canids, although these studies were not conclusive. In addition, the evidence presented from these studies consisted of drawings rather than actual images. However, their descriptions corresponded with the findings reported by Salazar et al. (1992) [99], who finally characterized the dog AOB using lectin histochemical studies.

In the meerkat, similar to canids, the AOB is difficult to identify macroscopically. However, microscopically, the pattern of lamination in the meerkat is more defined than that in dogs, resembling more closely the pattern described in cats, including well-defined glomeruli, a broad MPL with relatively numerous principal cells, and granular cells that are organized into clusters. This similarity was not unexpected because meerkats belong to the suborder Feliformia. However, the thickness of the layers, the degree of glomerular differentiation, and the number of cells found in the meerkat AOB appear to be lower than those observed in the cat. These differences remain to be verified through the performance of a rigorous morphometric study with a minimum number of specimens.

These observations become even more relevant in the context of the debate regarding the taxonomic classification of the meerkat. Its assignment to a family of Feliformia has oscillated between Herpestidae and Viverridae [28]. The meerkat is currently considered part of Herpestidae, along with a large number of mongoose species that share a wide range of social complexities with meerkats. The wide range of morphological diversity in the VNS has served as a fruitful tool for establishing phylogenetic relationships between different mammalian species and identifying evolutionary pathways [100]. Despite the advent of genomic techniques, which have made possible the population-level characterization of genetic variability at a resolution that was unimaginable until recently, the study of morphological characteristics remains a crucial element in the taxonomic classification of mammals. The VNS remains an important reference for such studies [101,102]. Future studies on the neuroanatomical and neurochemical characteristics of the VNO and AOB of the meerkat, combined with future observations in other Viverridae and Herpestidae species, may help to clarify this taxonomic debate.

### 4.2. Inmunohistochemical Study

Among the various immunohistochemical markers employed in this study, G protein labelling is of particular interest because it provides relevant information regarding the expression of VRs involved in the detection of sociosexual semiochemicals. G proteins are components of the cell-signaling cascades that are activated in VNNs [103]. The Gαi2 and Gαo subunits are associated with the two primary VR families, V1R and V2R, respectively [49,50]. The immunohistochemical analyses of G protein distribution in the AOB of several mammals initially demonstrated that V1R-Gαi2 neurons project to glomeruli located in the anterior region of the AOB, whereas V2R-Gαo neurons project to glomeruli in the posterior region of the AOB. This segregated projection has been described in Rodentia [104,105,106], Marsupialia [82,107], and Afrosoricidae [108]. However, this is not a uniform feature among mammals, as the Gαo pathway has deteriorated in several orders, including Carnivora, such as in dogs [59], cats [90], and foxes [109]; Artiodactyla [110]; Perissodactyla; Insectivora; Primates [111]; Hyracoidea [112]; and some Rodentia species, such as squirrels [112].

Our results regarding the expression of the G protein subunits Gαi2 and Gαo in the AOB align the meerkat with the group of mammals that have lost the expression of the V2R family. In the MOB, the expression patterns for both G proteins coincide with those observed in the mouse and in other studied mammals, presenting the ubiquitous expression of Gαo in virtually all glomeruli and the deep layers of the MOB and the absence of anti-Gαi2 immunolabelling [113,114].

OMP is a specific marker for neurons that originate in the olfactory and vomeronasal neuroepithelia and reach the superficial layers of the entire bulb [115]. In the adult meerkat, anti-OMP immunolabelling was restricted to the nerve strata and glomeruli of the OB, with slightly weaker expression in the AOB than in the MOB, similar to Lagomorpha [116], Didelphidae [117], and Vulpinidae [109]. Anti-GAP-43 is an additional and very useful marker for discriminating between mature axons and regenerating nerve fibers because GAP-43 expression in newly formed fibers decreases rapidly after the fibers reach their postsynaptic targets. Thus, GAP-43 is thought to be associated with the processes of neurite outgrowth and synaptic plasticity [54,118]. The olfactory system is a region of the adult nervous system that continues to exhibit extensive synaptic plasticity; therefore, the identification of high levels of GAP-43 expression in the meerkat OB is not surprising and resembles similar observations reported for other mammals, including the mouse [119] and rabbit [116].

Anti– MAP-2 and anti–SMI-32 were employed to characterize the principal cells of the OB. MAP-2 is a major component of microtubule junction bridges in dendrites and serves as a useful marker for the dendritic arbors of mitral/main cells [120]. In the meerkat, MAP-2 was primarily detected in the MPL and GrL of the AOB, presenting with the dense labelling of small neuronal somas, consistent with the pattern described in the OB of the rabbit [116]. Anti–SMI-32 primarily labels neuronal cell bodies and dendrites [52]. In the meerkat OB, anti–SMI-32 labelling was observed in the MOB, presenting intense stating of the somas and prolongations of the principal cells of the mitral and plexiform layers, similar to the pattern observed in other mammals, such as mice [121] and pangolins [47]. By contrast, monotremes show no SMI-32 labelling in the MOB [122]. In the meerkat, no appreciable anti–SMI-32 labelling was observed in the AOB, similar to the pattern described in pangolins [47].

The glial cell marker GFAP showed a high level of expression in the meerkat OB, suggesting active neuron-glial interactions, which are known to be involved in synaptic communication, plasticity, and the dynamic monitoring of brain function in the adult brain [123]. In the MOB, the anti-GFAP immunolabelling was concentrated in the superficial layers, whereas in the AOB, the glial processes extended throughout the structure, and immunostaining could be observed for astrocytes in the MPL. Anti-GFAP can also be used in mammals and other vertebrates to identify the ensheathing that myelinate the nerve fibers of the VNL [124,125]. The density of the glomerular cell bodies and fibers stained with anti-GFAP in the VNL, GlL, and MPL of the meerkat AOB contrasts with the limited numbers of astrocytes identified in the dog AOB [91] and more strongly resembles the dense labelling observed in the rat [57], wallaby [82], and fox [109] AOB.

Calcium-binding proteins are neuroactive substances expressed in large quantities in the OB, often used to identify segregated neuronal populations and allowing for the neurochemical distinction of neuronal types. In the VNL of the meerkat AOB, the vomeronasal fibers were completely immunopositive for both CB and CR, producing a strong neuropil staining in all glomeruli. However, PG cells were not immunopositive for either CR or CB, which may be associated with the low number of PG cells found in the Nissl staining.

AOB principal cells showed no immunostaining for either calcium-binding protein, similar to observations reported for the opossum [80], mouse [56], rat [126], and African wild dog, although in AOB of the latter species only CR expression was studied [46]. Overall, the anti-CR staining pattern observed in the AOB meerkat was similar to those observed in the rat [126], opossum [80], and African wild dog [46], whereas the pangolin displays a higher density of cellular elements and fibers, especially in the granular layer [47]. Anti-CB labelling observed in the surface layers of the AOB revealed more significant interspecies differences than anti-CR labelling. In rats [127] and opossums [80,128], the neuropil was stained much less strongly than that in the meerkat, and in both rats and opossums, some individual glomeruli of the AOB were stained strongly and were delimited by isolated positively stained PG cells.

The anti-CR pattern of immunolabelling in the MOB glomeruli (Figure 11) revealed the striking and intense immunolabelling of PG cells; however, in the neuropil, the anti-CR staining intensity was very weak, except for isolated subpopulations of unrelated glomeruli located in the vicinity of the AOB, which showed strong anti-CR immunostaining. Based on their location, these atypical glomeruli might be involved in the processing of chemical signals from the VNN, as has been hypothesized for the mouse olfactory limb glomeruli [129]. The presence of atypical glomeruli in the MOB is not a novel finding, as some examples have been described in the past, such as the necklace complex in mice [130] or the subset of glomeruli with high acetylcholinesterase reactivity [131]. To our knowledge, this is the first time that an atypical glomerular complex has been characterized by calcium-binding proteins, although studies of the OB using anti-CR antibodies in opossum [80] and African wild dogs [46] have identified subpopulations of CR-immunopositive glomeruli in the MOB; however, these studies did not provide information regarding the topographical relationship of these glomerular structures with the AOB. The presence of this atypical organization in proximity to the AOB in the meerkat suggests that the integration of information from the VNO is more complex than previously thought and may involve structures beyond the AOB.

In addition to an atypical glomerular pattern, the meerkat MOB shows an overall lower density of reactive elements stained for both CB and CR than the domestic dog, a species that shows a very high density of these elements, especially in response to CR immunolabelling. However, the African wild dog shows a lower density of immunolabelling in the superficial layers of the MOB, which were the only layers described by Chengetanai et al. in their study of the African wild dog olfactory system [46]. The quantification study by Choi et al. [45] showed a remarkable decrease in the immunolabelling density in the dog with an increase in age, especially for anti-CB labelling, which could explain the differences in the labelling patterns reported for CB expression in domestic dogs, wild African dogs, and meerkats. The MOB of the pangolin shows a pattern of CB and CR expression similar to that of the meerkat, characterized by a high density of PG cells immunopositive for both markers and the slight positivity of mitral cells for CR. However, in the granular layer, the intensity of labelling is higher in the pangolin, particularly for CB [47].

### 4.3. Lectin Histochemical Study

We studied the carbohydrate expression pattern in the meerkat OB by employing histochemical labelling with three lectins. The functional significance of the sugar groups detected by lectins is large, as these carbohydrates are involved in neurite outgrowth, synaptic plasticity, signal transduction, and cell–cell interactions [132]. The expression of glycoconjugates in the OB is very heterogeneous and species-specific, with differences identified even between closely related species. Therefore, as previous studies have indicated, lectin histochemistry is a useful option for the specific characterization of the VNS, able to discriminate between anterior and posterior zones of the AOB [82], an assessment that should always be considered when characterizing the organization of the AOB.

UEA has been identified as a specific marker for the overall vomeronasal pathway (VNO, VNN, and AOB) in adult mice [79,133], whereas the lectin BSI-B_4_ is recognized as an excellent VNS-specific marker in both rats [63] and opossums [64]. However, UEA did not produce positive labelling in the OB of the meerkat. A similar negative staining pattern has also been described in sheep [134], rabbits [116], and roe deer [84], whereas in other species, such as rats [63] and pigs [134], UEA labels both the MOS and the VNS. In meerkats, BSI-B_4_ was immunonegative in both the AOB and MOB, similar to the pattern described in rabbits [116]. LEA, however, stained the nervous and glomerular layers of both the AOB and the MOB. This pattern is highly conserved, described in a wide range of species, including mouse [133], wallaby [82], fox [109], deer [84], sheep, and pig [134]. These results suggest that glycoconjugates are involved in the organization of the primary olfactory and vomeronasal pathways in meerkats; however, the basis for their detailed expression patterns remains poorly understood.

In summary, our exhaustive morphological characterization of both the MOB and the AOB of the meerkat indicates that this species displays a neuroanatomical organization consistent with the classification as a macrosmatic mammalian species. Specifically, our findings in the OB SVZ suggest that this species could be an interesting model for the study of adult neurogenesis in mammals. The AOB structural features show an intermediate pattern between the most-studied members of Carnivora dogs and cats. Neurochemically, the AOB follows the G protein expression pattern typical of Carnivora, characterized by the lone expression of V1R family VRs. The organization of the MOB and AOB revealed by using other immunohistochemical markers suggests subtle species-specific differences, such as the presence of CR-positive atypical glomeruli in the MOB, highlighting the importance of chemocommunication among this species.

Our observations should help to establish the morphological basis for precisely elucidating the roles played by olfaction and pheromonal communication in this iconic species. Further anatomical studies should include other associated structures of both the MOS and VNS, in particular the olfactory nucleus and the amygdala.

## 5. Conclusions

This is the first study to reveal the neuroanatomical features of the MOS and VNS in the meerkat (*Suricata suricatta*), an iconic mammal with a marked social hierarchy. Our observations demonstrate that the degree of lamination and development of the meerkat MOB corresponds to a macrosmatic mammalian species. The chemosensory specialization for the detection of pheromones, including the expression of the V1R VR family and the deterioration of the V2R receptor family, was also observed.

## Figures and Tables

**Table 1:** Lectins and antibodies employed. Species of elaboration, manufacturer, dilutions, catalog number.

Ab/Lectin	1st Ab Species/Dilution	1st Ab Catalogue Number	2nd Ab Species/Dilution (Catalogue Number)
Anti-Gαo	Rabbit 1:100	MBL 551	ImmPRESS VR HRP Anti-Rabbit IgG MP-6401-15
Anti-Gαi2	Rabbit 1:100	Sta Cruz SC-7276	ImmPRESS VR HRP Anti-Rabbit IgG MP-6401-15
Anti-OMP	Goat 1:400	Wako S44-10001	Horse 1:250 Vector BA-9500
Anti-MAP2	Mouse 1:200	Sigma M4403	ImmPRESS VR HRP Anti-Mouse IgG MP-6402-15
Anti-GAP43	Mouse 1:800	Sigma G9264	ImmPRESS VR HRP Anti-Mouse IgG MP-6402-15
Anti-GFAP	Rabbit 1:400	Dako Z0334	ImmPRESS VR HRP Anti-Rabbit IgG MP-6401-15
Anti-Calbindin	Rabbit 1:5000	Swant CB38	ImmPRESS VR HRP Anti-Rabbit IgG MP-6401-15
Anti-Calretinin	Rabbit 1:5000	Swant 7697	ImmPRESS VR HRP Anti-Rabbit IgG MP-6401-15
Anti-SMI32	Rabbit 1:20	ENZ-ABS219-0100	ImmPRESS VR HRP Anti-Rabbit IgG MP-6401-15
UEA-I	1:10	Vector L-1060	Rabbit 1:50 DAKO P289
LEA	20 μg/mL	Vector B-1175	Vectastain ABC reagent PK-4000
BSI-B_4_	100 µm/mL	Sigma L-2140	Vectastain ABC reagent PK-4000

## Data Availability

All relevant data are within the manuscript, and are fully available without restriction.
